# Supplementary material for: Predicting the failure of two-dimensional silica glasses
Source: Nat Commun. 2022 May 20;13:2820. doi: 10.1038/s41467-022-30530-1 (PMC9122924; doi:10.1038/s41467-022-30530-1)
Supplement: Supplementary file 1 — Supplementary Information [file 41467_2022_30530_MOESM1_ESM.pdf]

Supplementary Information

Francesc Font-Clos,[1†], Marco Zanchi,[1†] Stefan Hiemer,[2†], Silvia Bonfanti[1,3], Roberto Guerra[1], Michael Zaiser[2], Stefano Zapperi,[1,2,3*]

[1] Center for Complexity and Biosystems, Department of Physics,
University of Milan, via Celoria 16, 20133 Milan, Italy
[2] Institute of Materials Simulation, Department of Materials Science Science and Engineering,
Friedrich-Alexander-University Erlangen-Nuremberg, Dr.-Mack-Str. 77, 90762 Fürth, Germany
[3] CNR - Consiglio Nazionale delle Ricerche,
Istituto di Chimica della Materia Condensata e di Tecnologie per l'Energia
Via R. Cozzi 53, 20125 Milan, Italy

[*]To whom correspondence should be addressed; E-mail: stefano.zapperi@unimi.it
[†] These authors contributed equally.

Supplementary Note 1

We have three different set of parameters in the SVM analysis: The parameters for the symmetry functions (δ, μ), the affine strain ϵ and the hyper-parameter of the support vector machine. δ is chosen as 0.1 Å. μ lies between 0.2 Å and some upper bound μ_{up} in steps of 0.2 Å. Atoms 2.5 Å larger than the upper bound away from the central atom are neglected. μ_{up} as well as the affine strain ϵ are varied along a set of possible values listed in table **1** . The SVM is optimized with respect to its regularization parameter C and two kernels (linear, radial basis function). The investigated parameter choices for C and can be seen in Table **1** as well as the investigated parameter range of kernel width γ for the radial basis function kernel. To investigate whether feeding symmetry functions of the initial configuration has any benefits, we train models also with just the features from the affine transfomred state and compare them with models trained from features of the initial and affine deformed state.

For every combination of δ, μ, ϵ, the SVM hyper-parameter are optimized via five fold cross validation on the training set, retraining for the optimal SVM parameters and judge the final mode by its performance on the test set. We perform and 80/20 split to generate the training and test set (total samples 913/910/737 for disorder levels 0.2/0.3/variable). As SVM do not scale well computationally with the size of the training set, we have to down-sample the training set to perform the training. We perform two different subset selections. For the first subset we choose all atoms which are part of the first bond breaking and an equal number of atoms from the rest of the population thus creating a balanced training set. For the second subset we again choose all atoms which are part of the first bond breaking and add enough atoms from the remaining (unbroken) population to reach final size of 10000 training samples thus generating an unbalanced training set. The weights are adjusted to rebalance the training set.

The biggest difference in model performance can be seen for the different construction of the training set (Supplementary Figure **2** a, e and i) where the balancing leads to a shift from overall correct predictions to the percentage of captured plastic events. For samples of variance 0.2 it can also be seen that the optimal kernel for models trained on the unbalanced training set is always the radial basis function kernel. The other model parameters are less obvious in terms of model impact. Models trained on symmetry functions of the initial undeformed and the affine deformed state come closer to the desired case of all correct predictions, the differences are small to models trained just on the affine deformed state (Supplementary Figure **2** b, f and j). Different values of the affine strain seem not to have a clearly distinguishable impact on the model performance (Supplementary Figure **2** c, g and k). This makes sense as the affine strain infers the orientation dependence on the atomic neighborhood, but the actual value of the affine strain is arbitrary in this application case as every sample has the first bond break at a different strain. The upper bound for the calculation of the symmetry functions does not have a strong impact on the final model performance (Supplementary Figure **2** d, h and l). This is coherent with the literature where it was found that as long as the upper bound includes several neighbor shells, its influence on model performance quickly drops.[16]

Supplementary Figures

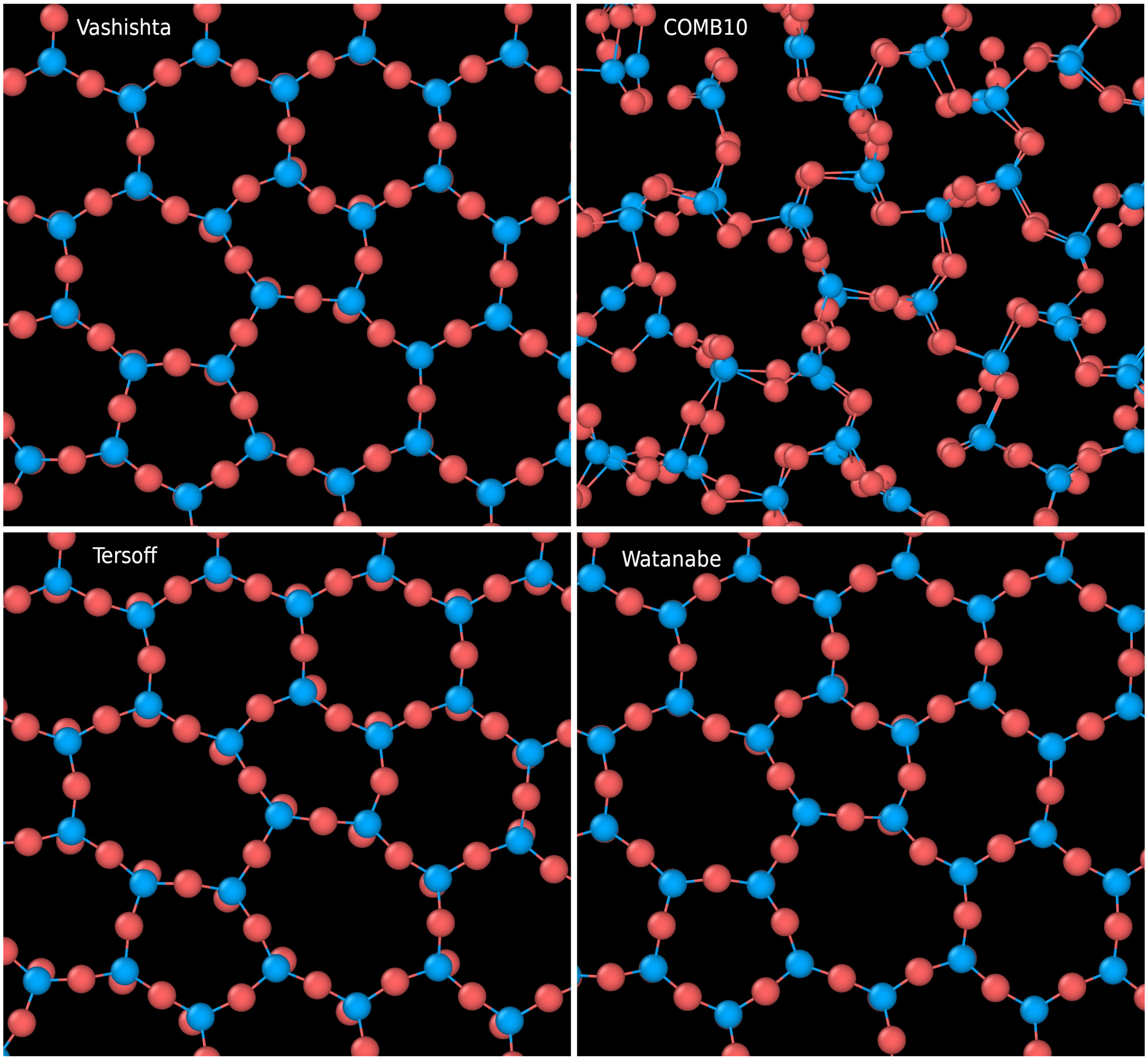

Supplementary Figure 1: Comparison of a configuration relaxed using different interatomic potentials.

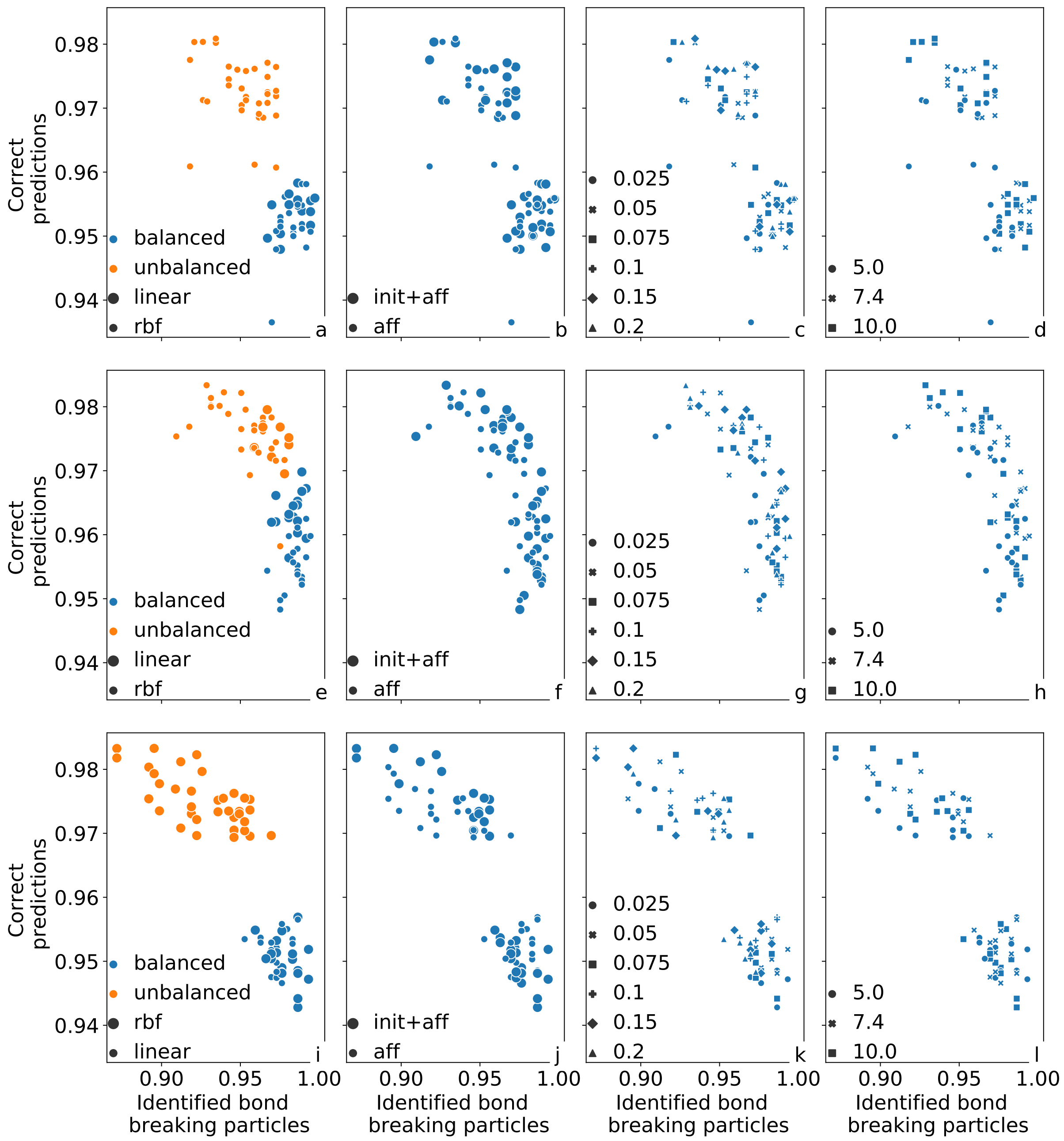

Supplementary Figure 2: Comparison of model hyper-parameters for disorder 0.2 (upper row), 0.3 (middle row) and variable disorder (lower row); a), e) and i) investigate different kernels with regards to differently constructed training sets; b), f) and j) highlight sensitivity with regards to the initial untransformed features; c), g) and k) show different strains used for the affine transformation; d), h) and l) examines the influence of the upper bound until which the symmetry functions are calculated.

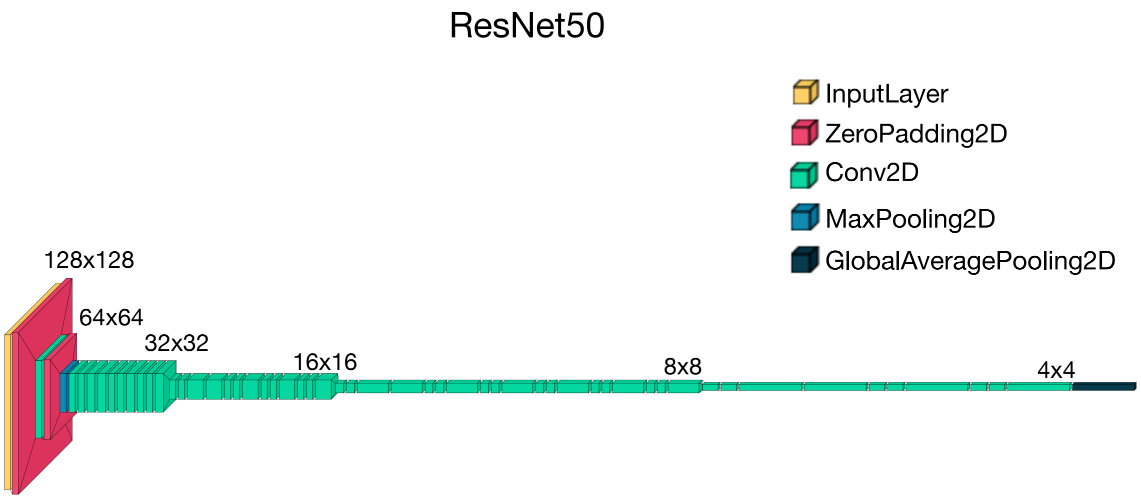

Supplementary Figure 3: **Scheme of ResNet50 architecture.** A scheme of Resnet50 architecture from Keras library has been obtained by kerasvisualization package. We have presented a schematic representation ignoring some layers. The input dimension is at first reduced to 64 pixels and then processed and subsequently reduced through the convolutional blocks (parts of ResNet50 architecture with the same dimension). ResNet50 can take as input any image with dimensions bigger than 32x32. The output layer of ResNet is a vector of 2048 dimensions (obtained by a global pooling of a tensor of 4x4x2048 dimensions) which as been passed in a fully connected layer to predict the disorder, the strain and the first bond break location. In our work we have considered the convolutional blocks with 32x32, 16x16, 8x8 and 4x4 dimensions.

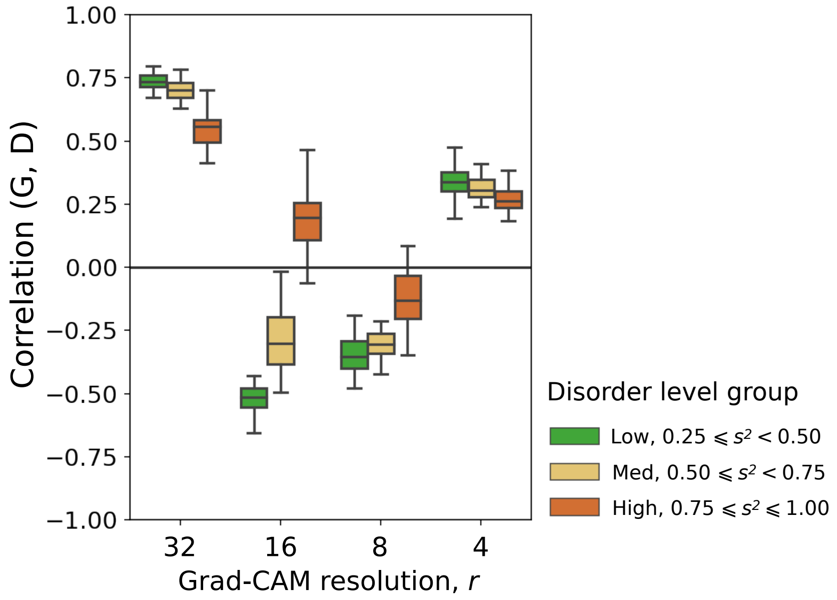

Supplementary Figure 4: Correlation between Grad-CAM attention values G and cell defects D in the variable disorder dataset $s^2 \in [0.25, 1]$. The Grad-CAM attention heatmap G is computed at four different resolution levels r. The panel shows that the Grad-CAM values G correlate positively with cell defects for $r = 32$ and $r = 4$, while for $r = 8, 16$ the correlation is negative. The disorder level spans three groups: low disorder ($s^2 \in [0.25, 0.5)$, green coloring), medium disorder ($s^2 \in [0.5, 0.75]$, yellow coloring), and high disorder ($s^2 \in [0.75, 1]$, red coloring), and shows that correlations are stronger in absolute value for low-disorder samples.

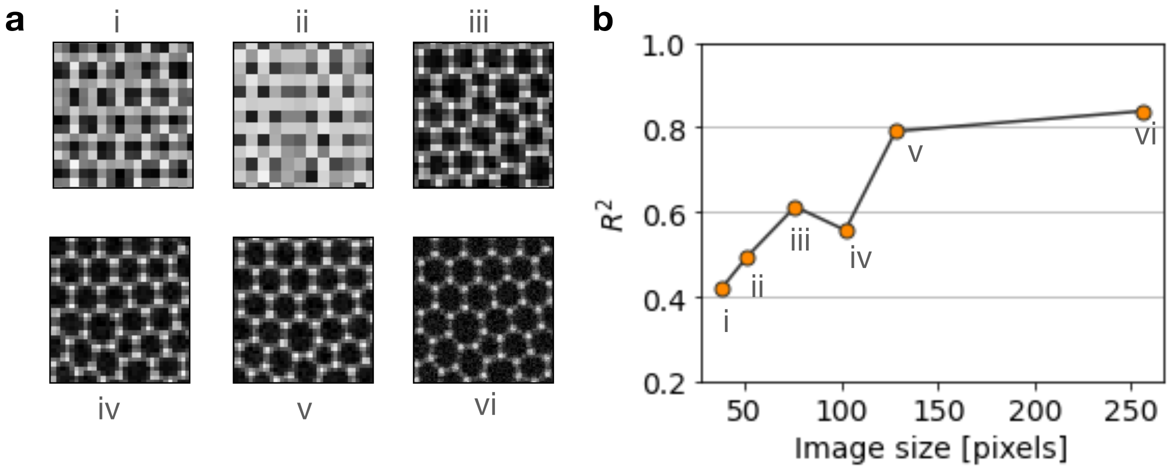

Supplementary Figure 5: a) Example of TEM-like generated image at different definition levels, from 36x36 pixels (i) up to 256x256 pixels (vi). The images show a subset of the full image only for clarity. b) R^2 coefficient of the strain-learning task as a function of the coarsening degree of the generated images. The panel shows that further increasing the coarsening beyond 128 pixels does not lead to better learning, as measured by the R^2 coefficient, while below 128 pixels the R^2 values sharply decrease. The roman numbers mark the corresponding example in panel A. We therefore use 128 pixel images along the rest of the manuscript. Training of the Resnet model for this figure included a dropout layer before the final dense layer with rate 0.2.

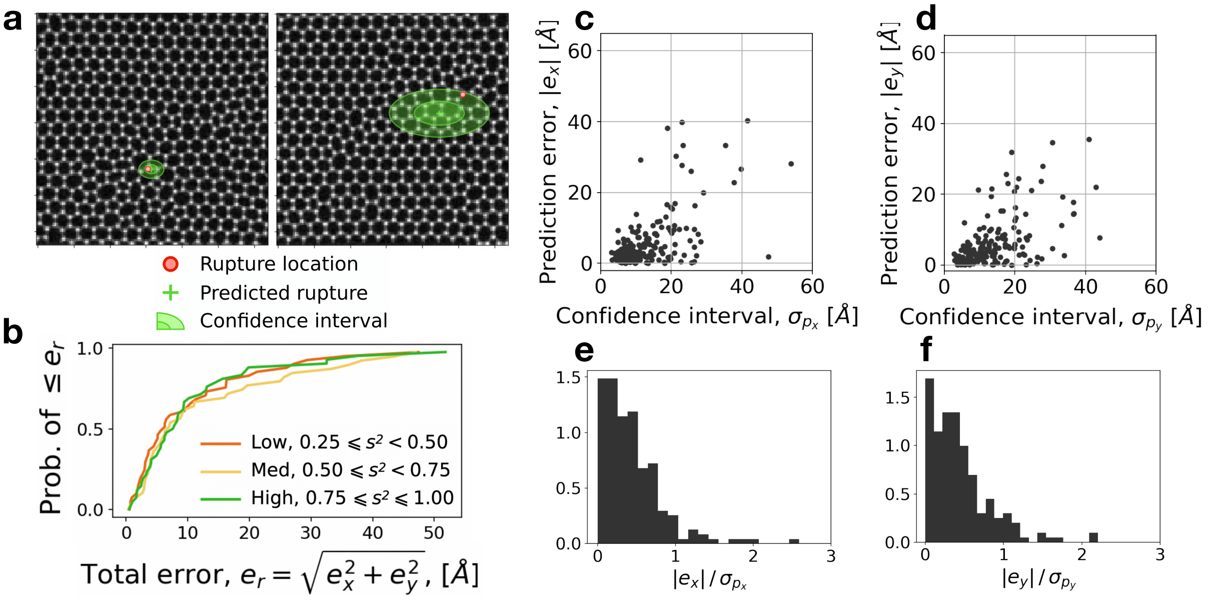

Supplementary Figure 6: **Error analysis of rupture location prediction.** a) Two examples of rupture location prediction. The model prediction (green cross), its confidence intervals (green ellipsis), and the real rupture location (red dot) are shown on top of two non-strained images used in the machine learning prediction. b) Cumulative probability of the total error e_r, for different disorder levels (see Methods for details). c, d) Scatter plot of the confidence intervals $\sigma_{p_x}, \sigma_{p_y}$, computed from the different predictions obtained from data augmentation, versus the absolute prediction errors $|e_x|, |e_y|$. The panels show that when the confidence interval is small, the prediction error tends to be small as well. e, f) Ratio of prediction error versus confidence interval, showing that for almost all samples the error is equal to or less than the confidence interval (one standard deviation of the predictions over data augmentation).

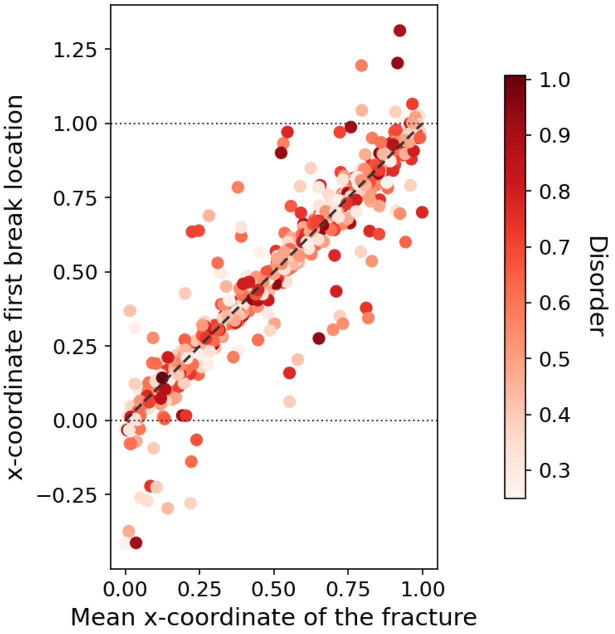

Supplementary Figure 7: **Correlation between crack path and rupture location.** Cross-correlation between the x coordinate of the first broken bond and the average x coordinate of the crack path. The color code represents the disorder s^2.

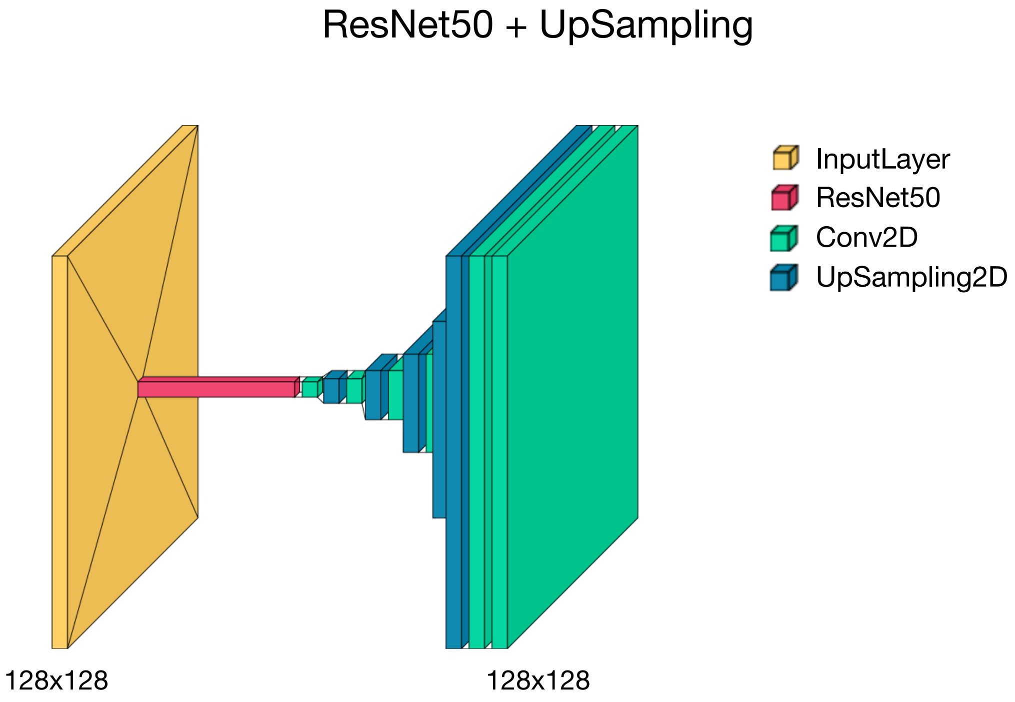

Supplementary Figure 8: **Scheme of ResNet50 architecture combined with upsampling layers.** A scheme of the architecture inspired by colorization model is presented. At first the input image is passed trough a ResNet50, which is discuused in Supplementary Figure **3**, producing a tensor of 4x4x2048 dimensions. This tensor is then passed through upsampling and convolutional layer until the input dimension is restored. This architecture allows to predict images. In our work we have used this architecture to predict the image of the fractured silica configuration.

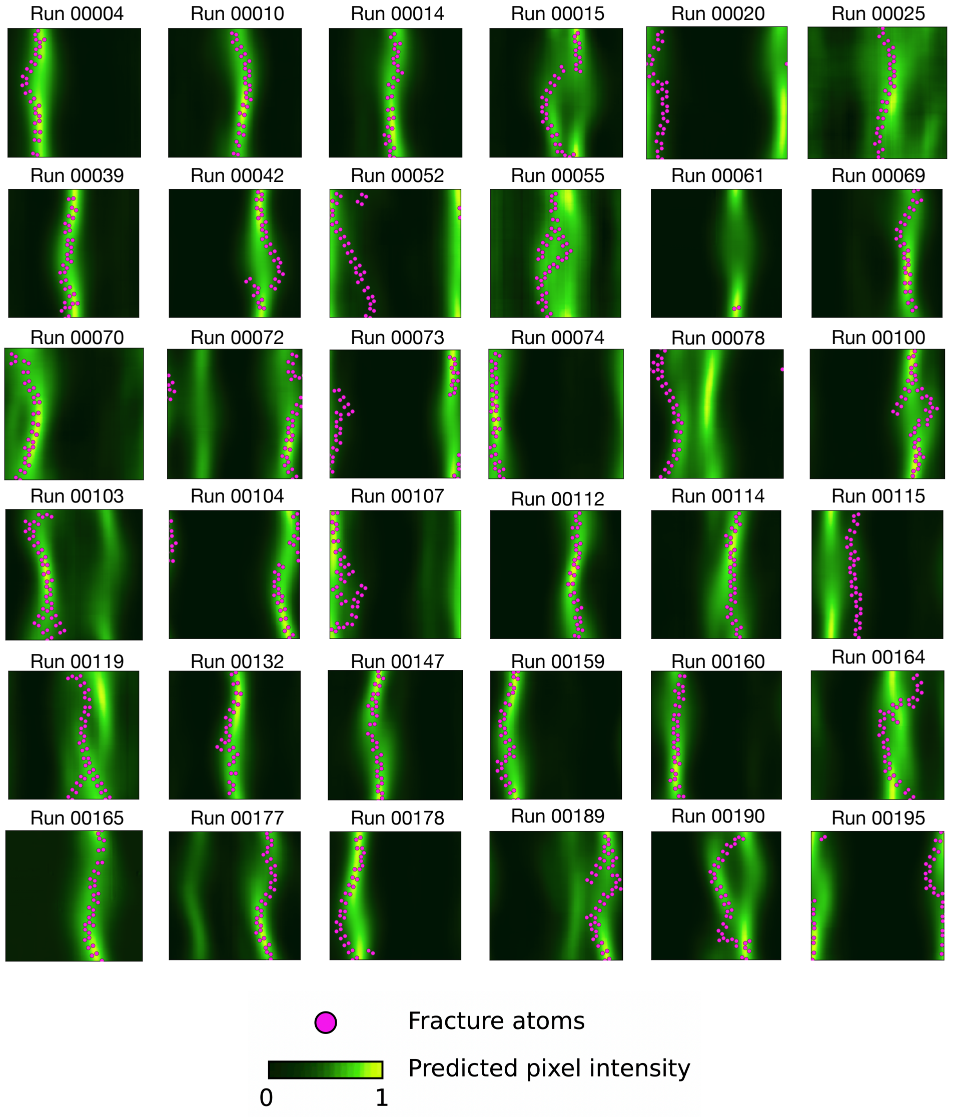

Supplementary Figure 9: **Crack path prediction.** The first 36 samples in the test set of the full crack path prediction task, see Supplementary Figure 5 for details (notice that samples are assigned to train/test sets randomly, not sequentially). For each panel, the fracture atoms are shown as magenta dots, and the model prediction as a black-to-green background, with greener area corresponding to more likely crack positions according to the model.

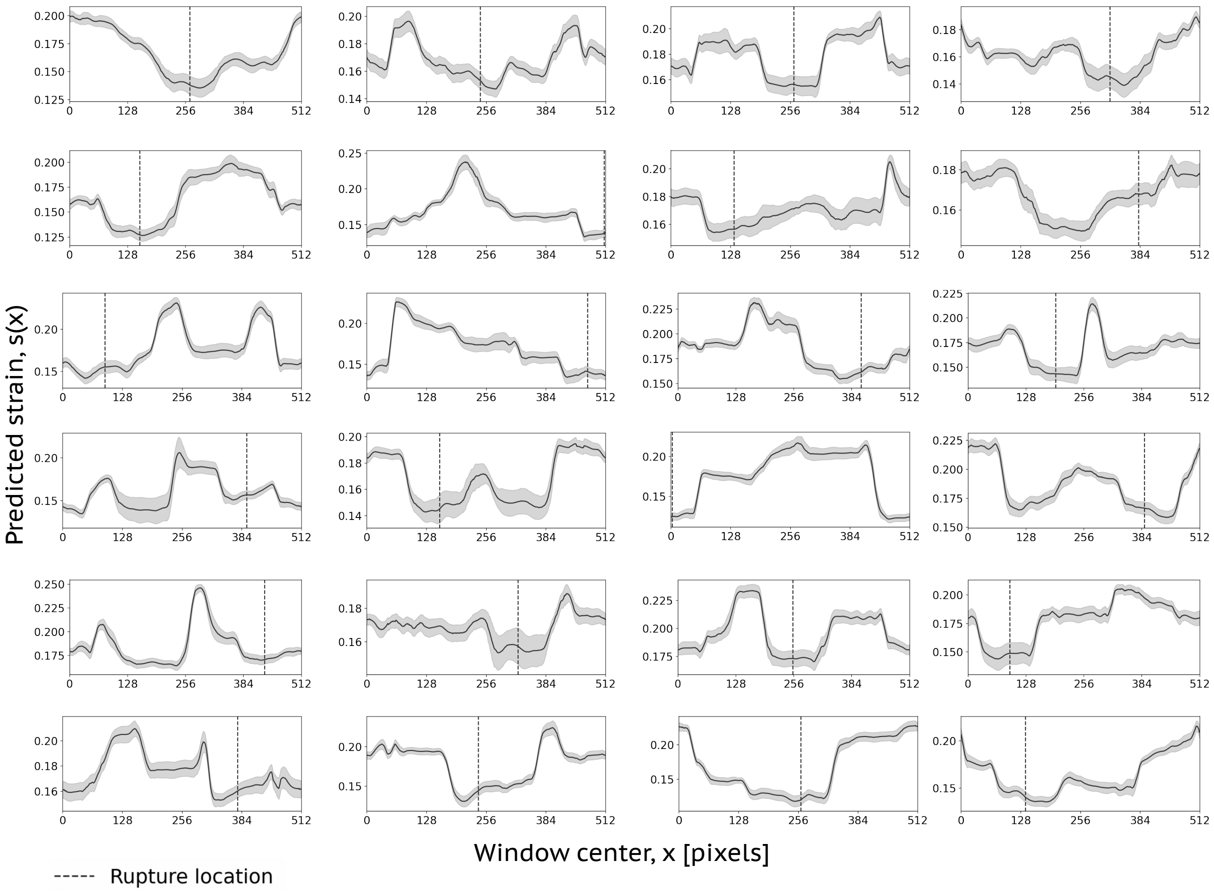

Supplementary Figure 10: **Transfer learning: predicting location from a strain-trained model.** First 20 samples of the strain-to-location task, where the strain-trained model is used to predict the rupture strain of different regions of a larger sample. By sliding a square window over different parts of the sample (horizontal axis), different rupture strains are predicted (vertical axis). The real rupture location is marked as a vertical dashed line. The figure shows that, in most cases, the rupture location tends to be on a region of lower predicted rupture strain.

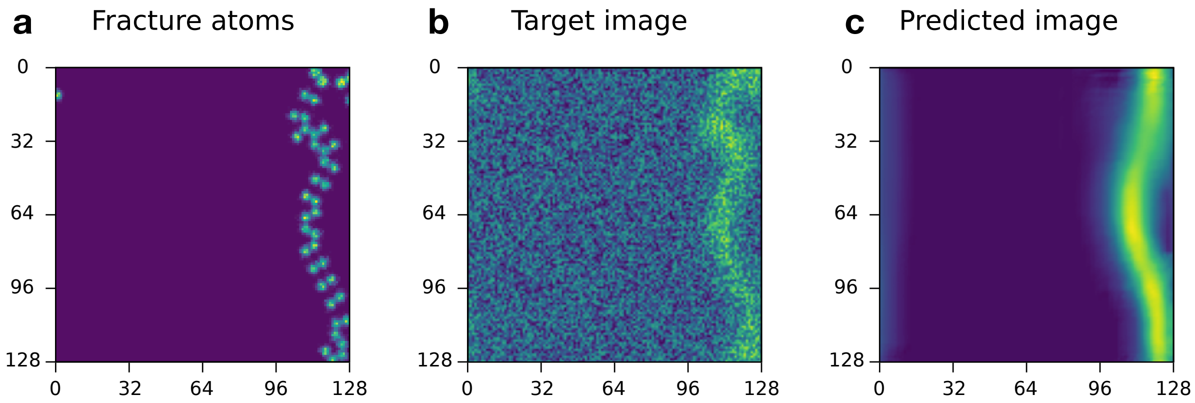

Supplementary Figure 11: **Crack path target construction.** a) Example of silica sample where only the atoms that are part of the crack are shown. b) Target image for the crack path prediction image-to-image algorithm. The show image is constructed starting from panel A and adding noise as detailed in Methods. c) Predicted image of the crack path prediction image-to-image algorithm. The algorithm correctly predicts the main shape of the crack.

Supplementary tables

μ_{up} [Å]	5.0, 7.4, 10.0
ϵ [%]	2.5,5,7.5,10,15,20
C	0.01,0.1,0.5,1.0,2.0,10,100
γ	1.e-05, 5.e-05, 1.e-04, 5.e-04, 1.e-03, 5.e-03, 1.e-02, 5.e-02, 1.e-01, 5.e-01, 1.e+00, 5.e+00, 1.e+01, 5.e+01, 1.e+02, 5.e+02, 1.e+03, 5.e+03, 1.e+04, 5.e+04, 1.e+05

Supplementary Table 1: Hyper-parameters of the symmetry functions and the support vector classifier which were investigated in this study.
